# Smart Release of the Antioxidant from Chitosan-Hyaluronan Reservoir in Skin Wound Healing †

**DOI:** 10.3390/pharmaceutics18050603

**Published:** 2026-05-14

**Authors:** Ladislav Šoltés, Tamer M. Tamer, Mohamed E. Hassan, Mojmír Mach, Katarína Valachová

**Affiliations:** 1 Centre of Experimental Medicine of Slovak Academy of Sciences, Institute of Experimental Pharmacology & Toxicology, Dúbravská cesta 9, 84104 Bratislava, Slovakia; ladislav.soltes@savba.sk (L.Š.); mojmir.mach@savba.sk (M.M.); 2 Polymer Materials Research Department, Advanced Technology and New Materials Research Institute (ATNMRI), City of Scientific Research and Technological Applications (SRTA City), New Borg El-Arab City 21934, Egypt; tmahmoud@srtacity.sci.eg; 3 Centre of Scientific Excellence-Group of Advanced Materials and Nanotechnology, Chemistry of Natural and Microbial Products Department, National Research Centre, El Behouth Street, Cairo 12622, Egypt; me.hassan@nrc.sci.eg

**Keywords:** antioxidant therapy, hydrogen atom transfer (HAT), hydrogel wound dressing, reactive oxygen species (ROS), single electron transfer (SET), smart biomaterials

## Abstract

Reactive oxygen species (ROS) play a dual role in biological systems as indispensable signaling molecules and as key mediators of oxidative damage. Chronic wounds are often associated with excessive ROS formation, which impedes the healing process. Locally applied antioxidants may mitigate such imbalance and support tissue regeneration. The chitosan–hyaluronan (Ch/HA) composite membranes’ biocompatibility, moisture retention, and ability to maintain local redox balance rank these materials among the most promising next-generation wound dressings. The aim of this review is to summarize the Ch/HA composite membranes serving as reservoirs for locally applied antioxidants to accelerate the healing of full-thickness skin wounds in experimental animals. Composite Ch/HA membranes fortified with the selected antioxidants such as phosphatidylcholine dihydroquercetin and glutathione were evaluated in rats. A mitochondria-targeted derivative such as mitoquinone mesylate was evaluated in rats and rabbits, while l-(+)-ergothioneine has been examined in rabbits so far. This review demonstrates that smartly designed Ch/HA composite membranes represent a novel-generation wound dressing platform capable of smart antioxidant release and efficient stimulation of skin regeneration.

## 1. Introduction

The earliest forms of life, archaea and bacteria, tolerated the Earth’s atmosphere dominated by volcanic gases such as a mixture of hydrogen, hydrogen sulfide, methane, carbon monoxide, and carbon dioxide. During the phylogenetic era, cyanobacteria metabolized/converted H_2_O molecules into hydrogen and oxygen gases. The gradually increasing concentration of O_2_ molecules in the atmosphere during the Precambrian period was reflected in a truly explosive expansion of aerobic prokaryotes and eukaryotes on Earth. Some animal species became extinct due to high concentrations of atmospheric oxygen, but those that adapted managed to survive. Aerobic metabolism is a complex system of endogenous processes that use oxygen molecules mainly to produce ATP (adenosine triphosphate), which is primarily the source of energy for life. Aerobic metabolism also generates reactive oxygen species (ROS), which are not only the major source of cellular damage but also serve as biogenic signaling molecules. The understanding of ROS homeostasis and cellular signaling pathways mediated by these species yielded the novel view of physiologists—finely regulated mechanisms [1]. The ROS, “by-products” of cellular aerobic respiration, are small neutral or charged molecules, such as superoxide anion radical (O_2_^●−^), hydrogen peroxide (H_2_O_2_), singlet oxygen (^1^O_2_), and the most reactive species hydroxyl radical (HO^●^) [2,3].

**Figure 1:**
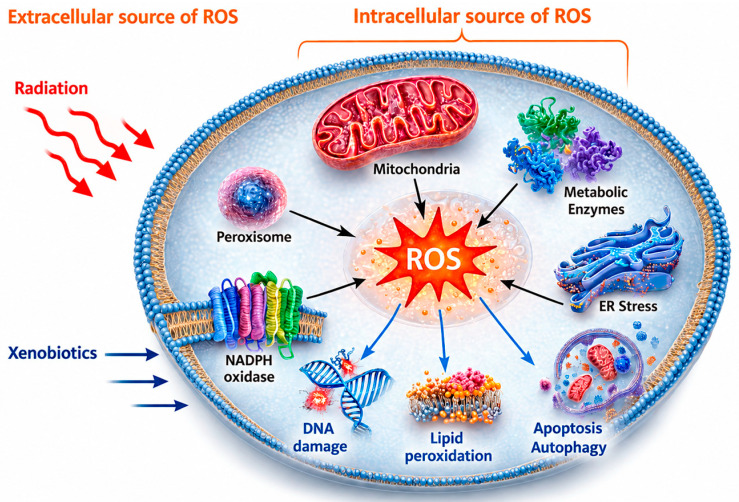
Schematic representation of oxidative stress induced by intracellular and extracellular stimuli. Extracellular sources of ROS include environmental factors such as radiation and xenobiotics. Intracellular ROS are generated mainly in the mitochondria, as well as other organelles such as the peroxisome and ER—endoplasmic reticulum (particularly the rough-ER), and in addition by some metabolic enzymes—nicotinamide adenine dinucleotide phosphate (NADPH) oxidases (adapted and modified from [1]), which catalyze the transfer of electrons from NADPH to molecular oxygen, producing O_2_^●−^, which can subsequently form H_2_O_2_ and hydroxyl radicals [10].

Oxidative stress is a pathophysiological imbalance between ROS production and the ability of cells to detoxify them. This imbalance can cause inflammation, tissue damage, and organ dysfunction. Understanding and managing oxidative stress is an important challenge for biomedicine. Cells use antioxidant enzymes (SOD—superoxide dismutase and catalase) and antioxidants to regulate ROS levels and prevent oxidative stress. Antioxidants include, e.g., vitamins C and E [4,5,6,7,8,9], the tripeptide glutathione (GSH), and even proteins complexing transition metal ions. Cells of higher animals are under constant pressure from extra- (high-energy radiation, xenobiotics) and intracellular stimuli generated especially by cellular organelles. A schematic representation of oxidative stress induced by intracellular and extracellular stimuli is in Figure 1.

Excessive ROS production can lead to peroxidation of cell lipids and damage to proteins, RNA, and DNA, resulting in autophagy and cell apoptosis [11,12]. ROS can damage proteins, e.g., thiol groups in proteins/enzymes can be oxidized to disulfide (-S-S-), S-glutathionyl (-SSG), S-nitrosyl (-SNO), and S-sulfenyl (-SOH) groups [11]. One of the primary biomacromolecules harmed by oxidative stress is DNA, which can undergo up to 10,000 changes per cell every day [13]. 8-Hydroxydeoxyguanosine is a widely monitored marker of DNA damage by oxidative species [14,15].

Under physiological conditions, ROS generated by the mitochondrial oxidative respiratory chain, ER, and NADPH oxidases can be efficiently neutralized by a complex regulatory network—Nrf2—the key transcription factor mediating the cell antioxidative response [16,17]. The neutralization reactions increase the synthesis of both cytosolic and mitochondrial SODs and catalase, as well as the key molecules such as GSH and NADPH. The latter acts in glutathione reductase catalytic conversion of oxidized glutathione (GSSG) to its reduced state (NADPH + GSSG + H_2_O ⟶ 2GSH + NADP^+^ + OH^−^) thus controlling the physiologically optimal GSH/GSSG ratio and thereby maintaining redox homeostasis in the cell [17,18]. The production of ROS is also critical to skin wound healing, contributing to platelet activation, antibacterial defense, and angiogenesis [19,20]. However, disruption of this finely tuned balance is closely related to the pathogenesis of a wide range of diseases [17,21] and is also associated with impaired wound healing, particularly in chronic wounds and metabolic conditions [19].

These findings provide a basis for investigating redox regulation as a mechanism for improving therapeutic strategies. While antioxidant-based therapies have been promising in early stages where oxidative stress plays a primary pathological role, their efficacy in diseases characterized by complex, multifactorial etiologies remains controversial. Yet, understanding the details of the complex relationship between oxidative stress and pathogenesis of diseases might optimize therapeutic strategies in clinical practice [22,23,24]. As Halliwell claimed, antioxidant therapy may help treat or even cure disorders where redox imbalance is the primary factor [25]. Broad-spectrum antioxidant therapies, however, may have negative side effects and fail to achieve therapeutic goals because of the complexity of redox signaling. Therefore, understanding the mechanisms of redox regulation in various human disorders and identifying specific drug targets or critical sites of modifications may provide new approaches to design precise therapies for difficult-to-treat diseases [26].

In severe cases, antioxidants can be administered by injection under medical supervision, which remains one of the most common delivery routes [27]. Another promising approach is transdermal delivery through the skin. Numerous biopolymers have been investigated as artificial skin materials, particularly those combining positively and negatively charged components, which can form stable matrices that function as both storage systems and carriers for antioxidants. An important advantage of this approach is the ability to bypass first-pass liver metabolism [28]. Among charged biopolymers, chitosan and hyaluronan are of particular interest. Chitosan is the primary choice as a positively charged biopolymer, while hyaluronan, its negatively charged counterpart, offers distinct advantages as a bioactive signaling molecule that regulates wound healing through strong receptor interactions and cellular signaling, unlike other negatively charged polysaccharides such as alginate or carrageenan.

A literature search was conducted in the following databases: ClinicalTrials.gov, Google Scholar, PubMed, Scopus, and Web of Science. The search strategy used the terms “chitosan-hyaluronan (Ch/HA) composite membrane”, “hydrogen atom transfer (HAT) and single electron transfer (SET) antioxidants”, and “skin wound healing” in the past 20 years, with prevalence in the last 5 years. The exclusion criteria were the following: purely cosmetic or anti-aging skin studies without wounds and dietary or nutraceutical applications of antioxidants.

This review evaluates Ch/HA composite membranes as smart biomaterial platforms for the localized and controlled delivery of antioxidants in skin wound healing, with particular emphasis on their ability to modulate the redox microenvironment, scavenge excessive reactive oxygen species, and accelerate tissue regeneration. A comparison of antioxidants operating through distinct redox mechanisms such as SET and HAT is summarized.

## 2. Reactive Oxygen Species and Methods Evaluating the Antioxidant Efficacy

The Weissberger biogenic oxidative system (WBOS) is highlighted as a significant generator of ROS. Moreover, the methodologies for assessing the protective effect of antioxidants against the degradative action of ROS—specifically oximetry and rotational viscometry—are discussed.

### 2.1. Weissberger’s Biogenic Oxidative System

Researchers primarily perform studies on the antioxidative effect of any (low-molecular-weight) compound in a test tube [29]. The essential components of the mixture in a test tube are:

A generator of ROS,
an indicator of the changes caused by ROS.

**Scheme 1:**
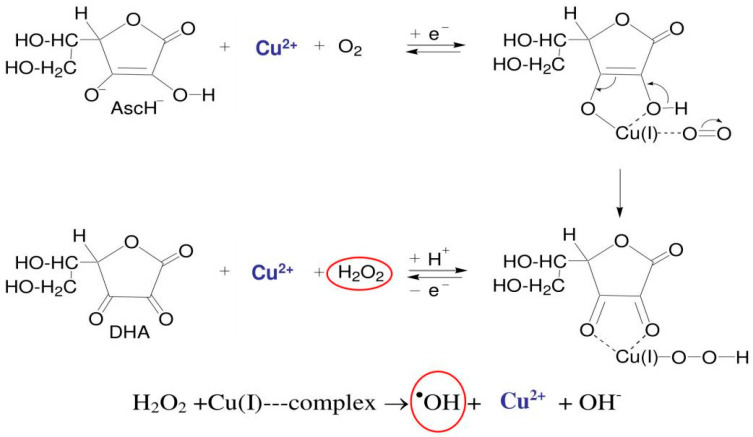
The Weissberger biogenic oxidative system. AscH^−^ and DHA are ascorbate monoanion and dehydroascorbic acid, respectively [29].

One of the progressive arrangements for studying the protective effect of xenobiotics is the use of WBOS, consisting of transition metal ions, particularly Cu^2+^ and ascorbate monoanion (AscH^−^), which under aerobic conditions generates primarily H_2_O_2_ molecules and simultaneously HO^●^ radicals (Scheme 1).

Along with reactions in Scheme 1, it is important to note that AscH^−^ simultaneously undergoes two consecutive redox reactions, resulting in the formation of a rapidly decomposing intermediate—the ascorbyl anion radical, which has been confirmed by electron paramagnetic resonance. This radical then dismutates, yielding ascorbate monoanion and dehydroascorbic acid [30].

### 2.2. Oximetry—Arrangement I

**Figure 2:**
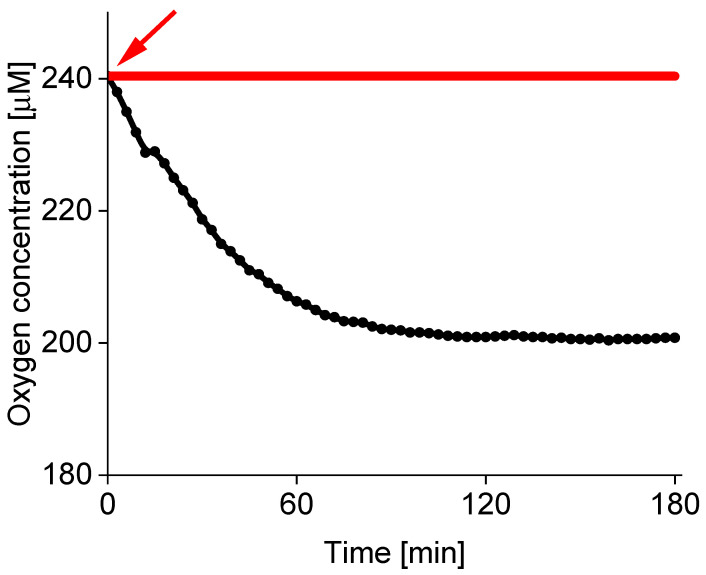
Changes in the concentrations of dissolved oxygen in the aqueous solution of 1 μM Cu^2+^ ions (CuCl_2_) and 100 μM ascorbic acid (black curve) monitored in the oximeter and those showing a hypothetically complete scavenged ROS by an antioxidant (red curve). The arrow indicates the time of addition of the antioxidant into the reaction vessel.

As shown in Scheme 1, during the above reaction sequence, 100 μM of ascorbate monoanion theoretically generates 100 μM of hydrogen peroxide, from which 100 μM of HO^●^ radicals are formed, while 100 μM of oxygen is consumed. Moreover, the red curve (cf. Figure 2) is a hypothetical result of using an appropriate amount of the investigated antioxidant with 100% protective effect. Nevertheless, it should be noted that 1 μM Cu^2+^ ions when sequestered with a complexation agent (e.g., isatin) might bring false conclusions. Thus, the detection of a curve between the pair of red and black curves demonstrates that the investigated substance scavenges HO^●^ radicals, most likely by donating a hydrogen atom.

**Scheme 2:**
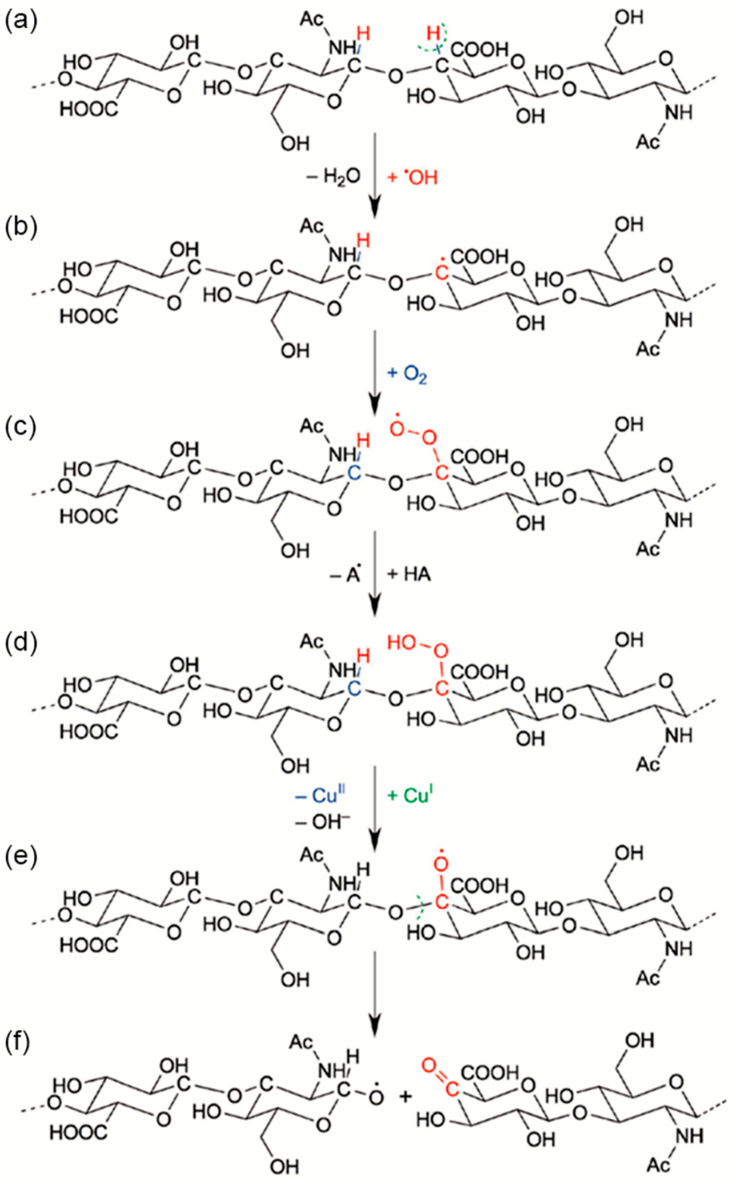
Free-radical hyaluronan degradation; initiation: (**a**) an intact HA macromolecule reacts with ^●^OH radical; (**b**) formation of an intermediate, i.e., a *C*-centered HA macroradical (denoted later A^●^). Reactions of propagation and of transfer of the free-radical center: (**c**) formation of an alkylperoxyl-type macroradical (in red); (**d**,**e**) generation of a HA hydroperoxide and a highly unstable alkyloxyl-type macroradical (in red). Reaction yielding fragments: (**f**) an alkyloxyl-type macroradical and an HA macromolecule bearing a terminal C=O group. Both fragments have reduced molecular sizes due to HA free-radical fragmentation reactions [32,33].

In the human body, hydroxyl radicals play a key role in the initiation and subsequent continuous propagation of free radical peroxidation of lipids in cells [31]. The fact that the microenvironment around the lipid molecule in the organism is made up of hydrophobic solubilizers may be one of the justifications for substituting a lipid (or more often its salt) as an indicator of the changes caused by ROS (cf. Section 2.1). Therefore, the free transfer of e^−^_(aq)_ (and/or H^●^) is most likely limited. To study the permanent peroxidation reaction in an aqueous environment, instead of any lipid, one could use an endogenous macromolecule, particularly high-molecular-weight hyaluronan [32]. Under such experimental conditions, the sequence of (radical) chain reactions is depicted in Scheme 2:

### 2.3. Oximetry—Arrangement II

After adding an appropriate amount of high-molecular-weight hyaluronan to the HO^●^ radical-producing WBO system, free-radical degradation of HAs was initiated (cf. Scheme 2 [33]). The HO^●^ radical, generated by the WBO system, in a sequence of chain reactions degrades HA in subsequent steps (b), (c), and (e), producing alkyl-C^●^, alkyl-C-OO^●^, and alkyl-C-O^●^ radicals. Therefore, the reaction mixture in the oximeter includes a variety of reagents that participate in a series of redox reactions (Table 1).

As evident from Figure 2, the consumption of oxygen molecules in the experimental setup described in Section 2.2 stops at a certain time. However, in the setup described in Section 2.3, further consumption of O_2_ continues due to reaction steps from (b) to (c) and ongoing free-radical degradation of biopolymeric species after reaction step (f). According to the theory of free-radical degradation of polysaccharides, such as hyaluronic acid, Scheme 2 illustrates that step (f) and the subsequent steps indicate the fragmentation of high-molecular-weight HA. A method of rotational viscometry can be used to readily monitor the steady decrease in the dynamic viscosity of the solution, which is functionally linked to the gradual decrease in the molecular weight of HA [34,35].

### 2.4. Viscometry—Arrangement I

**Figure 3:**
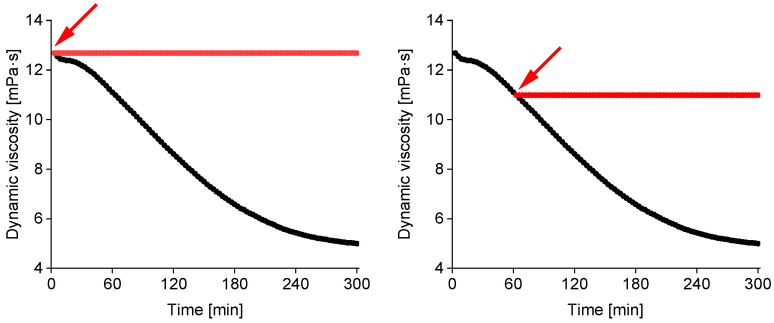
Time-dependent changes in dynamic viscosity of the HA solutions exposed to the WBO system composed of 1 μM Cu(II) ions and 100 μM ascorbate (black curve) monitored by rotational viscometry and the changes after the addition of an appropriately effective antioxidant at time 0 min, which hypothetically scavenged the reactive species generated by WBOS (red curve, (**left panel**)). Time-dependent changes after the addition of an appropriately effective antioxidant at time 60 min, which hypothetically scavenged the actual reactive species within the viscometry vessel (red curve, (**right panel**)). The arrow indicates the time of addition of the antioxidant into the reaction vessel.

Figure 3, left panel, black curve, illustrates the kinetics of the decrease in dynamic viscosity of a high-molecular-weight hyaluronan solution by the WBO system (the identical working solution used in Section 2.3). The red curve is a hypothetical record of the dynamic viscosity of the HA solution, where an antioxidant added at time 0 min completely prevented WBOS-induced HA degradation. As plausible, the area between the red and black curves demonstrates that the investigated antioxidant scavenges HO^●^ radicals, most likely by donating a hydrogen atom (cf. paragraph Section 2.2).

### 2.5. Viscometry—Arrangement II

Figure 3, right panel, black curve, represents the kinetics of the decrease in dynamic viscosity of a high-molecular-weight hyaluronan solution by the WBO system. The red curve is a hypothetical record of the dynamic viscosity of the HA solution where an antioxidant added at time 60 min completely prevented WBOS-induced HA degradation. Thus, the area between the red and black curves demonstrates that the investigated antioxidant scavenges alkyloxyl- and alkylperoxyl-type radicals, most likely by donating hydrogen atoms [cf. Scheme 2c,e]. Table 2 summarizes several compounds that have been investigated as scavengers of free radicals generated in vitro.

## 3. Structure and Physiological Functions of Human Skin

Skin, the largest organ of the human body, constitutes the primary protective barrier between the underlying tissues and the hostile action of the environment and against adverse noxious agents generated, e.g., by pathogens [41,42,43]. The skin in an adult human covers an area of approx. 1.5–2 m^2^, its thickness is in a range of 0.5–4 mm [44,45]. The slightly acidic pH value of 4.5–5.5 ensures that the skin surface prevents the growth of bacteria, yeasts, and fungi [46,47].

The skin has thermoregulatory, sensory, and immune functions, and protects against adverse harmful substances [48,49]. Since the skin is like a thick membrane, some compounds/drugs may readily enter the skin from top to bottom layers:

Stratum corneum: thickness 10–15 μm,
epidermis: 150 μm,
dermis: 1–2 mm,
hypodermis/subcutis.

The surface cells of the epidermis form the stratum corneum layer, consisting especially of keratin, a water-insoluble protein [50,51]. The epidermis continuously grows from cells in the deepest layer (the stratum germinativum), which lies immediately above the dermis [52,53,54]. The dermis consists mainly of fibrous connective tissue (composed of collagen fibers), blood and lymphatic vessels, smooth muscle cells, and nerve endings. Through its blood network, the dermis supplies the overlying epidermis with nutrients [53,55]. Under normal conditions the wounded skin begins to heal through five phases: hemostasis, inflammation, migration, proliferation, and remodeling [56].

Hemostasis, as the first phase of cutaneous wound healing, is responsible for stopping the bleeding and creating a temporary matrix. When platelets adhere to the exposed collagen at the wound site, a blood clot is formed [57,58]. Further, the inflammatory phase follows, which is the most critical and usually takes 2 to 5 days [56]. At first, neutrophils accumulate, which allows phagocytizing pathogens in the injured tissue. Later, macrophages are crucial for removing cellular debris and regulating the immune response. Growth factors and inflammatory cytokines regulate angiogenesis, cell migration, and the initial phase of tissue repair.

In the proliferation phase, granulation tissue is formed. Collagen, the primary component of the extracellular matrix (ECM) that creates the structural framework of the healing tissue, is produced by fibroblasts that migrate to the wound site. To create a strong circulatory network to support the developing tissue, endothelial cells stimulate angiogenesis. Reepithelialization is linked with the proliferation and migration of keratinocytes along the edges of the wound. These cellular processes are directed by transforming growth factor and platelet-derived growth factor, which promote ECM component synthesis, migration, and proliferation [57,59].

Remodeling (maturation) is the last phase of wound healing, during which the temporary matrix is replaced with a more functional and ordered scar tissue. Excess cells undergo apoptosis, and collagen fibers realign and mature [57].

In skin, lipid peroxidation maintains homeostasis by modulating the composition and organization of epidermal lipids, including ceramides and free fatty acids, thereby contributing to stratum corneum permeability and protection against environmental insults. In response to physiological ROS levels, lipid peroxidation also plays a role in adaptive responses to oxidative stress, activating enzymatic and non-enzymatic antioxidant defense mechanisms, including SODs, catalase, glutathione peroxidase, α-tocopherol, and β-carotene, which prevent excessive lipid oxidation, thereby preserving cellular function and structural stability [60]. On the other hand, these agents, when facilitating ROS production, also deteriorate endogenous antioxidant defenses, thereby enhancing lipid oxidation [61].

Furthermore, UV radiation, particularly UVA, interacts with skin chromophores to generate ROS, thus triggering the peroxidation of epidermal lipids and augmenting tissue damage. This leads to the formation of lipid radicals and their hydroperoxides, which primarily decompose into malondialdehyde and 4-hydroxy-2-nonenal, causing cytotoxic, genotoxic, and pro-inflammatory effects [60].

Administering an antioxidant maintains inflammation at a threshold level, and the use of antioxidants is the most appropriate modulator for smart intervention in whether inflammation subsides or progresses to chronic inflammation.

The skin is a complex tissue that is under stress when long-term hypoxia persists (a lack of oxygen) at the site of inflammation [62,63]. Restricting oxygen absorption to the site of injury is counterproductive because ROS production during tissue hypoxia is significantly higher than in normoxia [64]. The flux of highly reactive oxygen species may contribute to tissue damage so significantly that chronic or non-healing inflammation may result in the organism’s collapse [65]. The question is whether applying antioxidants is an efficient way to scavenge the adverse ROS generation at the site of inflammatory skin and whether antioxidant chemicals may successfully counteract chronic oxidative stress in the skin.

### 3.1. Topical, Intradermal, and Transdermal Drug Applications

When the given drug or antioxidant reaches only the uppermost layer of the skin, we refer to this as topical application. When the drug penetrates the layer interwoven with blood capillaries and is additionally distributed in the bloodstream throughout the body, it is called a transdermal drug delivery system [66,67]. A certain advantage of transdermal application is that the drug that has entered the bloodstream bypasses the liver during its first passage through the body, which, in the case of oral application of the drug, largely digests this agent as food [53,68,69,70]. People have been applying cosmetic and therapeutic substances to their skin for thousands of years. Artificial skin-wound dressings are commercialized either free of any drug/antioxidant or smartly releasing an active principle into the skin [71]. And considering the fact that the HO^●^ radicals can be scavenged primarily by antioxidants donating (e^−^_(aq)_ + H^+^)/the atom ^●^H [72,73], it is necessary to pre-evaluate in detail the possible consequences of applying a particular compound to inflamed skin.

### 3.2. Artificial Skins Serving as Drug Carriers

There are numerous papers related to artificial skin, which serves as a temporary matrix to enhance skin healing [74,75]. Ponte et al. [75] showed that electrospun nanochitosan/HA membranes rather than Ch/HA membranes accelerated the wound healing in rats over 21 days. Silvestro et al. [76] incorporated HA at concentration 5% to chitosan film, which resulted in enhanced adhesion and proliferation of fibroblasts onto the wound dressing and prevented *S. epidermidis* adhesion. However, most Ch/HA wound dressings have been fabricated as hydrogels. For example, Li et al. [77] showed that Ch/HA composite functionalized hydrogel enhanced healing of excisional wounds in mice over 14 days.

As well as, numerous studies documented the use of Ch/HA composite membranes and especially hydrogels loaded with an antioxidant as a protective and therapeutic agent for wound dressings. For example, in 2018 Šoltés et al. [78] patented the preparation of self-associating Ch/HA membranes as a carrier (reservoir) for an antioxidant penetrating mitochondria to treat acute or chronic wounds. Zhou et al. [79] fabricated taurine-loaded carboxymethyl chitosan/oxidized HA hydrogel with good biocompatibility, anti-inflammatory properties and enhanced healing of the wounds of diabetic rats. The mupirocin-gallic acid-grafted quaternized chitosan/oxidized HA hydrogels had antibacterial effects and accelerated wound healing in a full-thickness skin of *S. aureus* infection mouse wound model [80]. Liu et al. [81] prepared Ch/HA/gold/fibroblast growth factors hydrogel, which had antibacterial effects and was supposed to be a suitable biomaterial for the treatment of diabetic wounds. Foroozandeh et al. [82] fabricated nylon 6/Ch/HA nanofibrous composite membranes (mats) with good physical-chemical properties, nontoxicity to fibroblasts and antibacterial effects. Salva et al. [83] showed that Ch/HA hydrogel prevented a rapid clearance of the loaded honey, thereby prolonging the healing effect of honey in full-thickness wounds in rats. In another study sodium hydrosulfide-loaded cerium oxide (NaSH@CeO_2_) was synthesized and incorporated into Ch/HA hydrogel, producing NaSH@CeO_2_/Ch/HA, which in the methicillin-resistant *S. aureus*-infected rat wound model achieved 98.1% wound closure rate by day 14 [84].

The advantages of applications of Ch/HAs wound dressings are as follows: they have non-toxic, biocompatible, biodegradable, bioadhesive, bioabsorbable, antimicrobial, hemostatic, and anti-inflammatory characteristics. The amino groups in chitosan have pK_a_ values of approx. 6.5, thus in neutral solutions/gels (pH 7) the macromolecules are positively charged [85,86,87]. *N*-Acetylglucosamine (NAG) units present in chitosan are dominant constituents of dermal tissue that are crucial for repairing scar tissues in addition to their contribution to the anti-inflammatory properties of chitosan. Furthermore, NAG promotes fibroblast proliferation and collagen synthesis. Chitosan plays an important role in thrombosis and blood coagulation, and it also stimulates the expression of cytokines and growth factors that promote angiogenesis, which is crucial for accelerating tissue restoration [88,89,90,91]. One of the most prominent applications of Ch/HA wound dressings are drug delivery, where they enable controlled and targeted drug release, protect encapsulated agents from degradation, and improve permeation across biological barriers [92].

Hyaluronan is the major component of the epidermis and dermis of the skin [92,93,94,95]. Hyaluronan, due to its hydrophilicity and biocompatibility, is widely used in the form of films, hydrogels, fibers, nonwoven textiles, and sponges [94,96,97,98,99]. Thus, HA is broadly used as an excellent moisturizer in cosmetics and skincare products [100,101,102]. Due to the presence of d-glucuronic acid groups, HAs in aqueous solutions/gels behave like polyanions; the overall pK_a_ values of HA-carboxyl groups are approximately in the range of 3–4 [103,104]. High-molecular-weight HAs exhibit strong bacteriostatic activity [105,106], which is primarily related to physical/interfacial properties of the polymer such as high hydration, steric exclusion, reduced surface fouling, and impaired adhesion/biofilm formation [107]. In wound healing, HA renders temporary support in transporting nutrients during increased migration and proliferation of keratinocyte cells [105]. Additionally, HA regulates the expression of fibroblast genes for the remodeling step and dominates the angiogenesis [105,108].

While chitosan itself forms a firm film when its slightly acidic solutions dry (usually in 2% aqueous acetic acid) [109,110], films made of HA without a chemical modification are softer, weaker, and more brittle. Hyaluronan readily absorbs water and loses structural integrity [110,111]. For this reason, when the solutions of chitosan and hyaluronan are blended in the right ratios, a viscous solution is formed. Then, such a solution readily dries to form a flawless thin film with significantly more tear resistance than a film made solely of the chitosan solution.

As mentioned above, most studies are focused on the fabrication of Ch/HA hydrogels. To our knowledge it is the first time we have evaluated the efficacy of membranes composed of Ch/HA membranes that are loaded with an antioxidant. The principle of smart release of antioxidants or drugs is that the carrier, which is composed of HA and chitosan in a ratio that provides a slight surplus of negative charge, can loosely bind a positively charged compound, i.e., it is opposite in polarity to the carrier.

When the composite membrane is applied to the damaged tissue, the positively charged compound gradually releases from the membrane into the site of action due to the compound concentration gradient between the membrane surface and the target site, as well as the mutual electric attraction between the membrane and the compound. Such composite membranes are used for the treatment of superficial chronic tissue damages, i.e., for the treatment of difficult-to-heal wounds [112].

To provide a desired shape and to strengthen the composite membrane, it is advantageous to impregnate a sparsely woven fabric/gauze or even the whole bandage with a viscous solution.

## 4. Single Electron Transferring (SET) Compounds

**Scheme 3:**
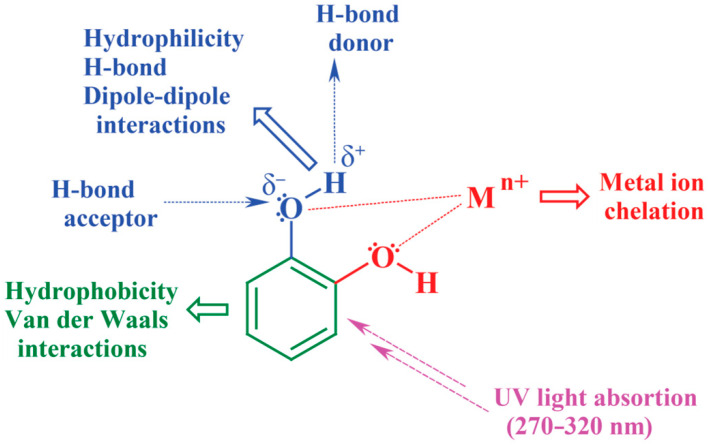
Basic physicochemical properties and reactivities of the phenol functional group (adapted with a copyright permission from Quideau et al. [113]).

The single electron transfer is one of the redox reactions by which, e.g., plants protect their DNA against exo-ROS. Protective SE-transferring substances, such as polyphenols, are often classified as plant secondary metabolites [113,114,115]. Due to a basic phenolic functional group, polyphenols’ intrinsic physicochemical properties have various benefits in both parent plants and other living organisms (Scheme 3). Concerning skin wound healing, a Ch/HA membrane is a reservoir for a SET compound whose antioxidative effect is linked to neutralizing free radicals by donating a single electron, thereby transforming ROS into a stable, non-radical species.

The phenyl hydroxy group (PhOH) forms an amphiphilic moiety that combines a hydrophobic group, i.e., a planar aromatic nucleus, with a hydrophilic polar hydroxy substituent, which, depending on the pH value, can act as a hydrogen bond donor or an acceptor. Hydrophobic van der Waals/π-stacking interactions and the formation of hydrogen bonds are seemingly dichotomic. At least two adjacent hydroxy groups on a phenyl ring facilitate metal ion chelation.

Moreover, compared to the secondary (π → π*) absorption maximum, the phenol is “red” and often further shifted to the absorption maxima within the UVB light range (270–320 nm); hence, the phenolics provide protection against DNA-damaging solar radiation.

Over five hundred different polyphenols with their common name, molar mass, chemical formula, the substance content in foods, their physico-chemical parameters, biochemistry and pharmacology, etc., are listed in phenol explorer http://phenol-explorer.eu/compounds (accessed on 29 April 2026) [116]. It is well-known that some compounds present in plants, including those of polyphenolics, coexist as synergists.

Below are several main classes of polyphenols with the number of items within each class:

Phenolic acids; 108

Flavonoids; 279

Stilbenes; 10

Lignans; 29

Other polyphenols; 80

Whereas each class has several subclasses, e.g., flavonoids include anthocyanins, proanthocyanidins, flavonols, flavones, flavan-3-ols (catechins), flavanones, and isoflavones [115,117,118].

**Figure 4:**
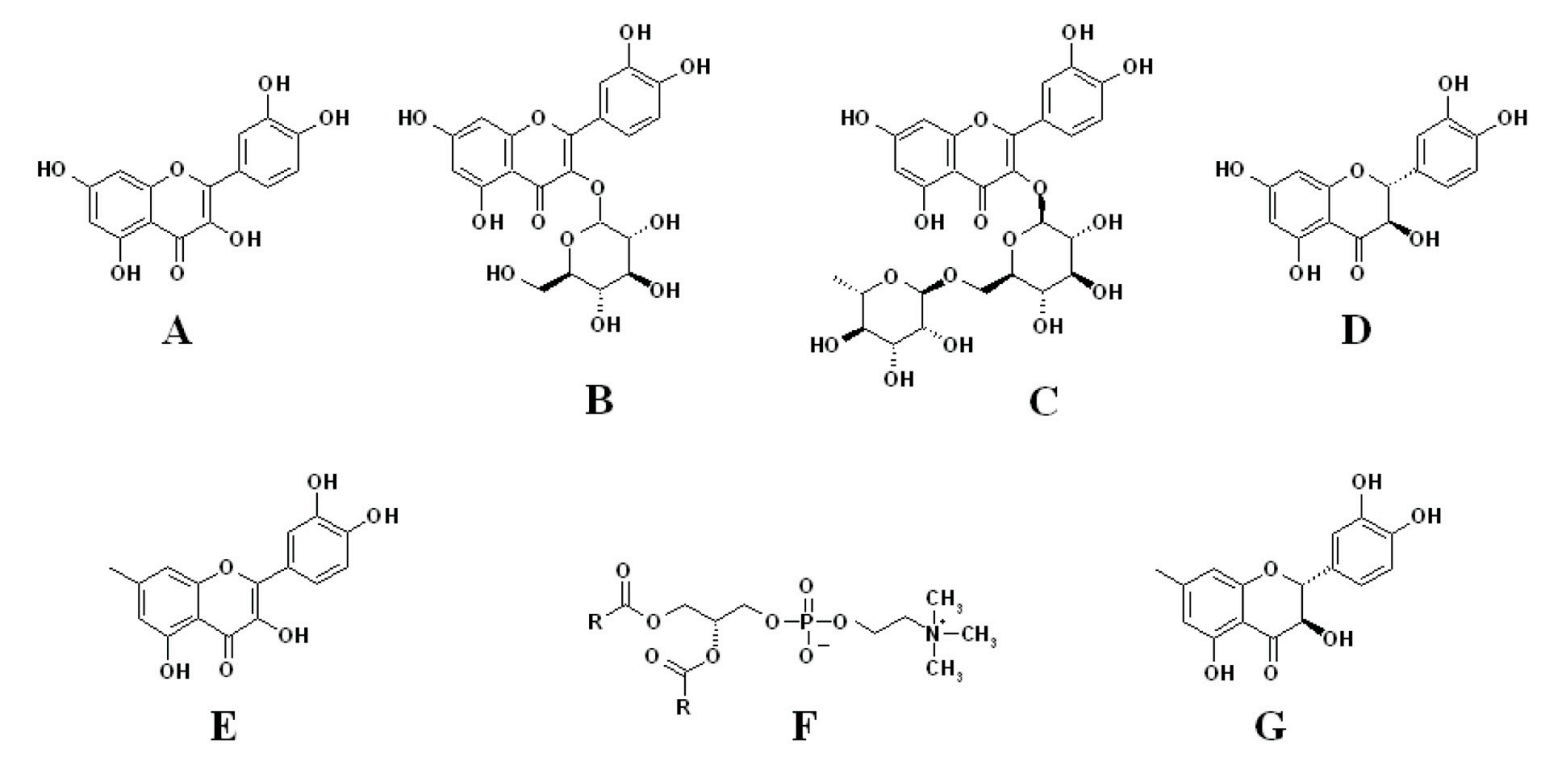
Chemical structures of (**A**)—quercetin, (**B**)—quercetin 3-*O*-glucoside (isoquercetin/hirsutrin), (**C**)—quercetin 3-*O*-rutinoside (rutin), (**D**)—dihydroquercetin (taxifolin), (**E**)—quercetin residue in phosphatidylcholine quercetin, (**F**)—phosphatidylcholine di-ester residue, and (**G**)—dihydroquercetin residue in phosphatidylcholine dihydroquercetin (PCDQ). (**F**); phosphatidylcholine di-ester residue is a zwitterion forming either liposome or oil/water and water/oil emulsions.) [123,124,125,126].

Currently, there are over 4000 known flavonoids. More often they occur as *O*-glycosides with a saccharide component, which allows increasing the hydrophilicity of flavonoids, e.g., glucose, galactose, rhamnose, arabinose, xylose, or rutinose. The degree of glycosidation generally has little effect on the antioxidant properties of flavonoids [119,120]. The plant-based polyphenols, such as quercetin with its three derivatives (two are *O*-glycosides) and the phosphatidylcholine derivatives [121,122] are as examples presented in Figure 4, while some properties of these compounds are listed in Table 3.

As deduced from Figure 4 and Table 3, the incorporation of *O*-glycoside moieties into quercetin led to an increase in the solubility of quercetin-*O*-glycosides in water. On the other hand, the *O*-glycoside derivatives emulsified and thus solubilized the water-insoluble quercetin (moiety) in a hydrophilic environment. At the same time, however, both quercetin-*O*-glycosides (Figure 4B,C) retained their SET properties, which resulted in the preservation of their actions/biological effects (see column 6 in Table 3). Similarly, the incorporation of two hydrogen atoms into the quercetin structure, i.e., the formation of the taxifolin molecule, partially increased the hydrophilicity of this compound (log P = 0.95) compared to the practically zero solubility of quercetin in an aqueous environment (0.01 mg/mL). At the same time, it is necessary to favorably evaluate the preparation and use of original compounds such as phosphatidylcholine quercetin and phosphatidylcholine dihydroquercetin (cf. Figure 4E,F).

Polyphenols loaded into the self-associating Ch/HA matrix must have the main characteristics as follows:

- A valuable therapeutic level,
- the affinity of the drug to the skin must be higher than to the delivery system,
- the minimal toxicity and allergenicity of the drug to the skin during long-term application.

The Lipinski rule of 5′ can be mentioned, as it predicts that drug distribution in the body, including the penetration of drugs through cell membranes, is governed by specific postulates [133], which in polyphenolics might be partly modified as follows:

- Chemical structure should have maximally 5 donors of hydrogen bonds,
- chemical structure should have maximally 10 acceptors of hydrogen bonds,
- chemical structure should not exceed a molecular weight of 500 Da,
- chemical structure should not have a partition coefficient in octanol/water (logP) lower than 5,
- chemical structure should not have more than 10 rotatable bonds.

Concerning polyphenols, there are the so called WBSSH criteria claimed by White, Bate-Smith, Swain, and Haslam [113,134,135], which are as follows:

- The molecular weight in the range of 500–4000 Da,
- generally moderately water-soluble compounds,
- possessing >12 phenolic hydroxyl groups,
- 5–7 aromatic rings per 1000 Da,
- The chemical structure should not have more than 10 rotatable bonds.

Therefore, despite the poor fit of phosphatidylcholine dihydroquercetin to the Lipinski rule of 5′ predictions, this substance nearly meets the WBSSH criteria. Given that orally ingested plant-based nutraceuticals are primarily distributed into the liver, where numerous enzymes alter or metabolize the parent substances, the topical skin application of antioxidative phosphatidylcholine dihydroquercetin could be a significant advancement.

Antioxidant and antimicrobial wound dressings are the most favorable for chronic wound treatment. The two self-associating biopolymers, chitosan and HA, have been prepared and patented [78,136] multifunctional polyelectrolyte wound dressing that can serve as an artificial skin (Ch/HA membrane).

### 4.1. Phosphatidylcholine Dihydroquercetin

**Figure 5:**
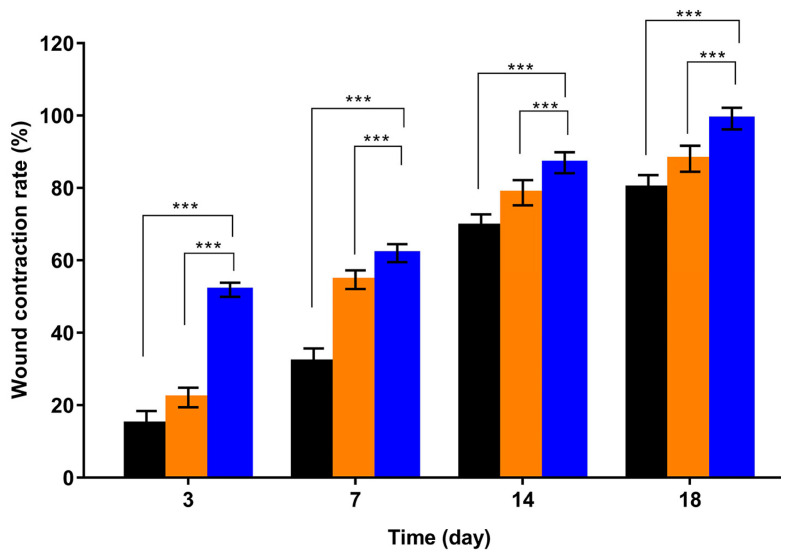
Wound closure rate vs. time in the rats: the untreated group (control, black), the group treated with the Ch/HA membrane (orange), and the group treated with Ch/HA/PCDQ membrane (blue). Data are presented as means ± SD, *n* = 5. Statistical analysis was performed using one-way ANOVA followed by Tukey’s post hoc test, *** *p* ≤ 0.001 compared with the control group [123].

To our knowledge, this is the first time the Ch/HA membrane has been loaded with a suitable antioxidant, e.g., PCDQ liposomes, to prepare a medicinal composite Ch/HA/PCDQ membrane. Figure 5 illustrates the wound healing in adult Wistar albino female rats with full-thickness wounds around 1.5 × 1.5 cm created on the dorsal area of rats’ skin on day 0. Quantitative wound closure was assessed by digital planimetry [123].

Three wound dressing efficacies were investigated: (i) cotton gauze serving as the negative control, (ii) the Ch/HA membrane, and (iii) a three-component composite membrane Ch/HA/PCDQ. As is evident from the results in Figure 5, wound healing during the first 3 days showed a remarkable difference. While the wound treated with cotton gauze was only 15.0 ± 0.75% healed, the use of the Ch/HA membrane led to healing to an extent of 22 ± 1.1%. When using the composite membrane containing PCDQ, the wound was healed to a value over 50% (52 ± 2.6%). This enhancement was likely due to the ability of the membranes to maintain a moist environment at the wound site, which is essential for effective healing [123,137]. Moist wound conditions support the migration of fibroblasts and keratinocytes and promote autolysis of necrotic tissue through controlled exudate absorption [138,139]. The active principle—PCDQ—in composite membranes, as discernible, lessened the wound area associated with the formation of dry scab three days post-operation, thus diminishing the duration of the inflammation process. This beneficial effect may stem from the capability of PCDQ to scavenge the excess ROS secreted by neutrophils and macrophages during the inflammatory stage, preventing the rise of oxidative stress that hampers the wound restoration. On day 18, the injuries treated with the PCDQ-containing composite membrane showed a wound contraction of almost 100%, while the Ch/HA group exhibited a wound contraction level of 88 ± 4.4%, surpassing the cotton gauze group by 8%.

The study demonstrated that the use of Ch/HA/PCDQ composite membranes significantly accelerated wound healing (in full-thickness rat skin), and furthermore, histological examinations of wounds treated with Ch/HA/PCDQ membranes documented significant reepithelialization compared to wounds treated with cotton gauze as well as single Ch/HA membranes. These findings demonstrate that composite membranes containing a suitably selected antioxidant have significant potential for wound healing and skin regeneration. The in vivo results depicted in Figure 5 demonstrated significantly accelerated wound closure and tissue regeneration compared to controls. The wound contraction rate for the Ch/HA/PCDQ group (over 52% on day 3 and almost 100% on day 18) was high [123].

### 4.2. Mitoquinone Mesylate

**Figure 6:**
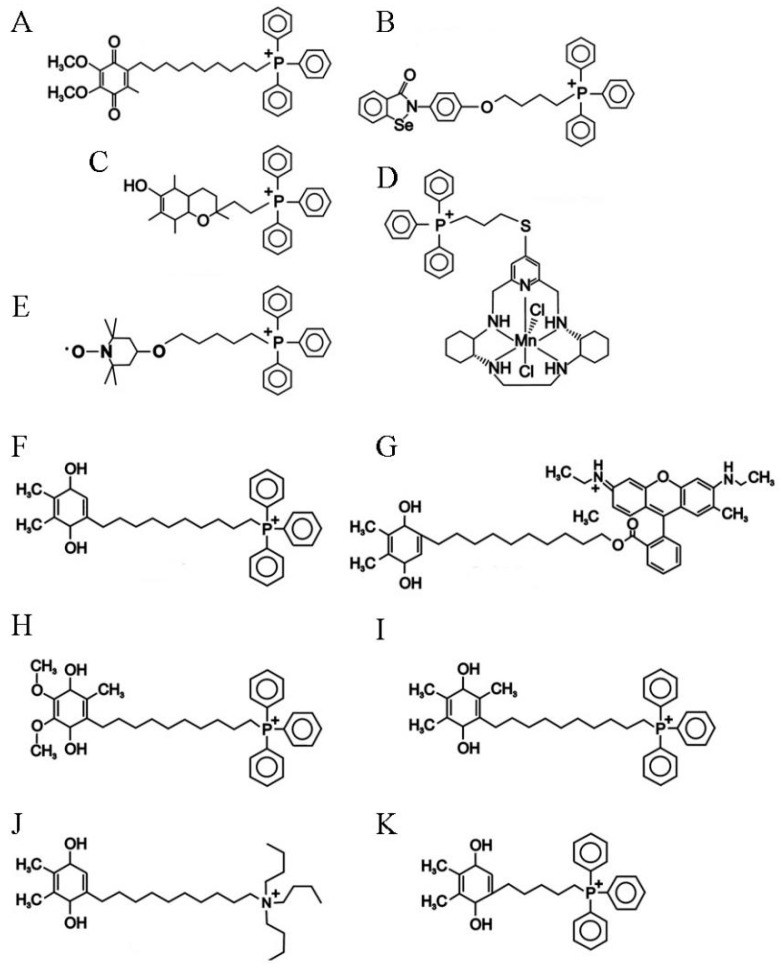
Chemical structures of several mitochondrially-targeted antioxidants: (**A**)—Mitoquinone mesylate (MitoQ, an antioxidative analogue of coenzyme Q10), (**B**)—MitoPeroxidase, (**C**)—MitoE_2_, (**D**)—MitoSOD, (**E**)—MitoTEMPOL, (**F**)—SkQ1H_2_, (**G**)—SkQR1H_2_, (**H**)—MitoQH_2_, (**I**)—SkQ3H_2_, (**J**)—SkQ4MH_2_, (**K**)—SkQ5H_2_ (adapted with a copyright permission from Gruber et al. [142]).

Intra- and transdermally acting antioxidative compounds that perform their ROS protective/healing function by penetrating the cell wall as well as drawing into mitochondria can be classified as mitochondria-targeting antioxidants (MTAs) [140,141]. Several MTAs, which bear a cation (usually P^+^ or N^+^) of “Skulatchev type”, are already commercially available (cf. Figure 6).

According to Figure 6, the positively charged MTA molecule is quickly drawn to the negatively charged cell surface by the cation(s) P^+^ or N^+^. After MTA enters the cell, the antioxidant molecule is directly drawn into the mitochondria, where it scavenges ROS [143,144,145]. However, if MTA enters a cell whose function is to generate the biogenic signaling ROS, the signal is extinguished. This can happen because biogenic ROS are essential signals that regulate cellular processes, and MTAs are designed to neutralize both harmful and signaling ROS. This suggests that the use of a mitochondrially targeted antioxidant may in some cases be counterproductive. For example, Doughan and Dikalov [140] showed that MitoQ may act as a prooxidant and has proapoptotic effects in endothelial cells since its quinone group can participate in redox cycling and production of O_2_^●−^ during forward electron transport in mitochondria respiring on the complex I. These results were also confirmed by Fink et al. [146]. Moreover, MitoQ acted as a pro-oxidant by producing H_2_O_2_ in isolated rat liver mitochondria [147].

**Figure 7:**
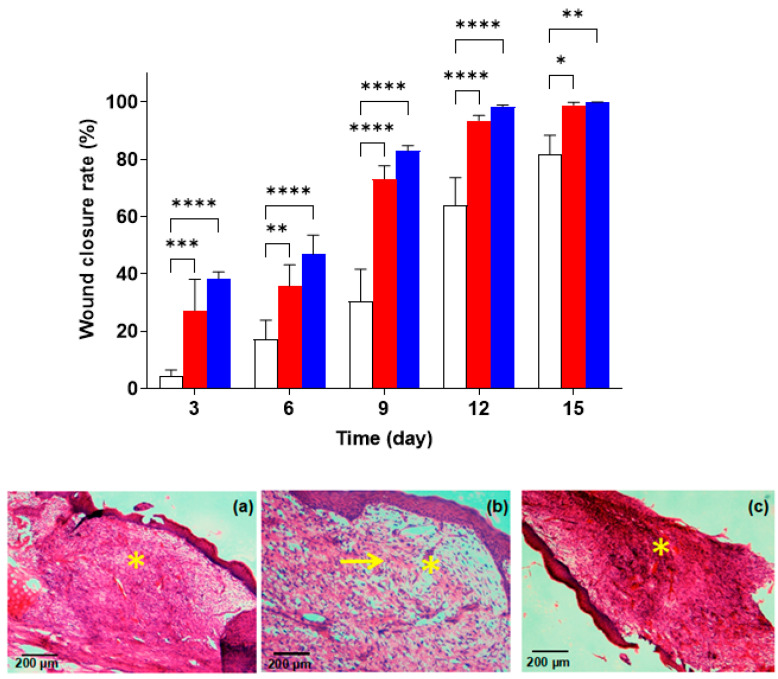
Wound closure rate vs. time in the ischemic rabbits: the untreated group (control, white), the group treated with the Ch/HA membrane (red), and the group treated with Ch/HA/MitoQ membrane (blue). Data are presented as means ± SD, *n* = 6. Statistical analysis was performed using two-way ANOVA followed by Tukey’s multiple comparison test, * *p* ≤ 0.05, ** *p* ≤ 0.01, *** *p* ≤ 0.001, **** *p* ≤ 0.0001 compared with the control group. Histology of skin wounds in ischemic rabbits after 15 days of treatment: (**a**) the untreated control group; (**b**) the group treated with Ch/HA membrane; (**c**) the group treated with Ch/HA/MitoQ membrane, asterisk—granular tissue, arrow—site of activated fibroblasts and collagen fibrils (adapted with a copyright permission from [148]).

The study by Tamer et al. [148] reported for the first time healing of wounds sized 1 × 1 cm in ischemic ears of male rabbits HIL (2.5 ± 0.5 kg) using the compound MitoQ, which could help design preclinical experiments involving human volunteers. As shown in Figure 7, upper panel, the results of measuring the wound area after three days showed only a slight reduction in the control/untreated animals. On the contrary, in the rabbits treated with Ch/HA and composite Ch/HA/MitoQ membranes, the expectation of significantly faster wound healing was confirmed. On day 6, a significantly faster reepithelialization of the skin wounds was visually confirmed compared to the control group. In the untreated animals, the percentage of wound closure on day 15 reached 81%. In contrast, in the treated animals the percentage of wound healing was over 90%, whereas in some animals skin wounds were completely healed and reepithelialized. The fastest reepithelialization was clearly recorded in wounds covered with Ch/HA/MitoQ composite membranes.

Figure 7a shows vascular granular tissues in the control/untreated group of rabbits after 15 days (marked with asterisk). Histological examination of wounds in animals in which Ch/HA membranes were applied, as indicated by the arrow in Figure 7b, revealed a likely site of activated fibroblasts with the formation of collagen fibrils. In the place marked with asterisk newly formed veins can be observed. In the group of rabbits treated with Ch/HA/MitoQ composite membranes, the area marked with asterisks (cf. Figure 7c) shows nonspecific granular tissue in the initial stage with enhanced cellularity. As mentioned above, the wounded skin begins to heal through five interdependent and overlapping phases: hemostasis, inflammation, migration, proliferation, and maturation [148,149]. In contrast, patients with chronic skin wounds usually need specialized treatment to help the healing process, depending on the type of lesion, its location, depth, and their condition [150].

**Figure 8:**
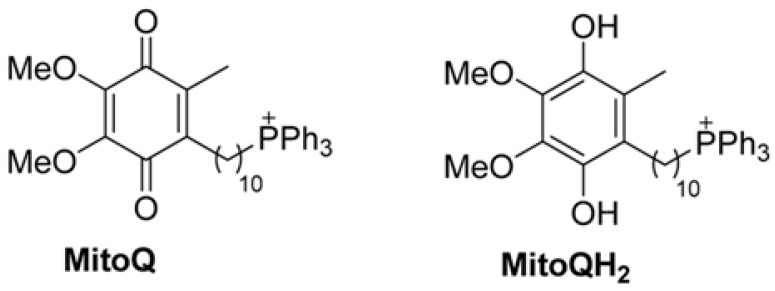
MitoQ (mitoquinone) and MitoQH2 (mitoquinolone) structures (adapted from Cheng et al. [147]).

When applying a composite membrane containing MitoQ (charge P^+^), it is reasonable to argue that the appropriate ratio of both charged biopolymers will ensure controlled and sustained migration of the charged drug to the membrane surface and from its surface to the affected skin. Penetration of MitoQ into skin cells and subsequently through the outer and inner mitochondrial membranes was most likely achieved. In mitochondria, MitoQ molecules energetically eliminate ROS by a redox—quinone ↔ hydroquinone—type reaction (Figure 8). Table S1 (Supplementary Materials) illustrates differences between SET and HAT mechanisms, and Table S2 summarizes several SET compounds, including MitoQ, that are still of interest in research, especially in the fields of cancer and cardiovascular and neurodegenerative diseases.

## 5. Hydrogen Atom Transferring (HAT) Compounds

**Figure 9:**
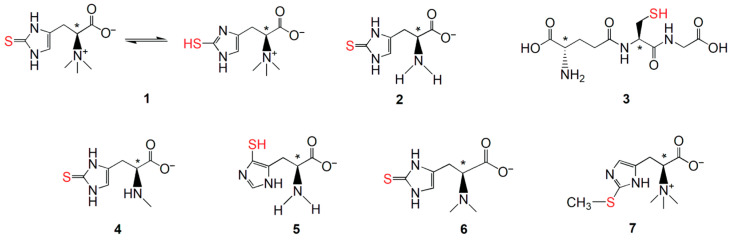
Structures of thione (**left**) and thiol (**right**) form of l-(+)-ergothioneine (**1**), l-2-thiol-histidine/nor-trimethyl-ergothioneine (**2**), l-glutathione (**3**), nor-dimethylergothioneine (**4**), nor-methyl-l-ovothiol A (**5**), nor-methyl-ergothioneine (**6**), S-methyl-l-ergothioneine (**7**) [151,152]. The asterisk (*) represents a chiral carbon.

Table S1 summarizes that numerous compounds, which provide a hydrogen atom to the counter-partner in redox reactions, are currently in clinical trials. This table also includes studies aimed at treating diseases from a redox perspective by using antioxidant compounds that transfer one or more electrons. These hydrogen atom-transferring compounds are H^●^ atom donors. The hydrogen in their molecule is relatively weakly bound (cf. thiols in Table 3, e.g., tiopronin [34], glutathione [39]; cf. Figure 9).

### 5.1. Glutathione

Glutathione is an endogenous antioxidant produced in the cytosol of almost all cells, especially in hepatocytes, epithelial cells, immune cells, red blood cells, and astrocytes to protect them against reactive oxygen species. Moreover, GSH plays an essential role in many intracellular functions, e.g., in reductive processes for proteins such as folding and maintaining stability, glutathionylation, and regulation of translation [153,154]. In DNA synthesis, GSH serves in the reduction of ribonucleotides, the repair of DNA single-strand breaks, the direct interception of DNA radicals, and as a redox sensor for the cell cycle [153]. In addition, it has physiological functions for storing and transporting cysteine. It acts as a coenzyme in several reactions with foreign compounds. The cellular concentration of GSH is around 1–10 mmol/L, while its concentration in plasma is only 1–3 μmol/L [155].

**Figure 10:**
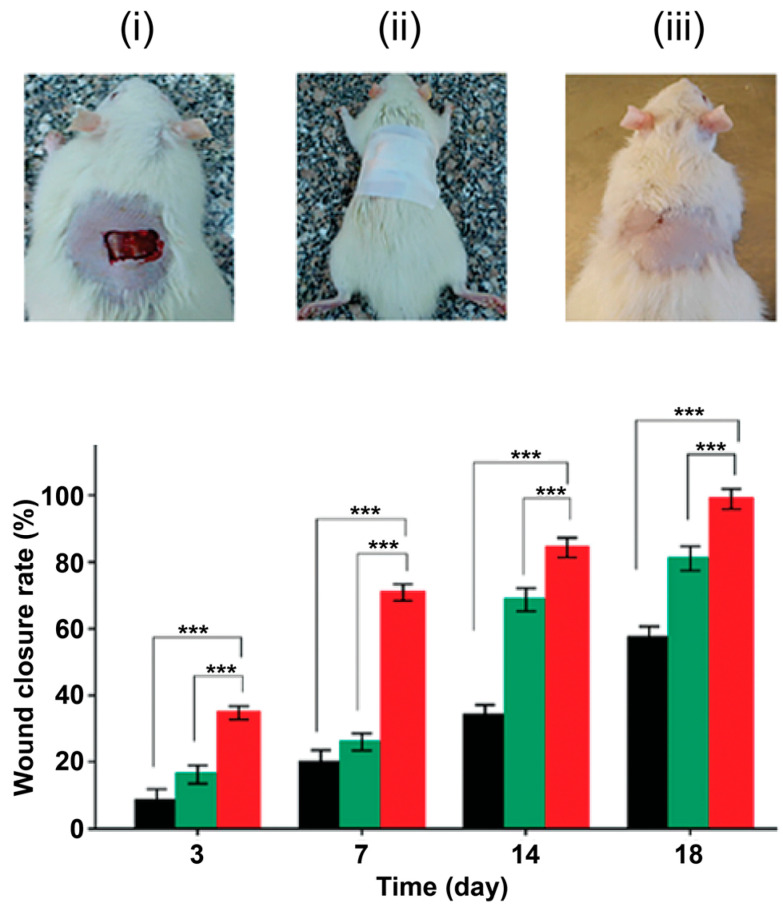
Wound closure in rats: (**i**) on day 0; (**ii**) the beginning of wound healing; (**iii**) the wound on day 18 of the treatment. Wound closure rate vs. time in the rats: the untreated group (control, black), the group treated with the Ch/HA membrane (green), the group treated with Ch/HA/GSH membrane (red). Data are presented as means ± SD, *n* = 5. Statistical analysis was performed using one-way ANOVA followed by Tukey’s post hoc test, *** *p* < 0.001 compared with the control group [39].

Figure 10 shows the effects of different dressings on wound closure: (i) the untreated Wistar male rats serving as the negative control, (ii) the Ch/HA membrane, and (iii) the Ch/HA/GSH membrane.

As shown in the results presented in Figure 10, the wound closures on day 3 were reported as follows: 8.4 ± 0.42% for the control group, 16.2 ± 0.81% for Ch/HA membranes, and 34.9 ± 1.74% for composite Ch/HA/GSH membranes. Visual observation when changing the closures within 3 days recognized that wounds in the control and Ch/HA groups were swollen with extrusions, while the Ch/HA/GSH group was dry. Wound healing achieved on day 7 showed a remarkable difference: While the wounds treated with Ch/HA/GSH composite membranes were partially healed with wound closures at the level of 71 ± 3.6%, the applied Ch/HA membranes and control group treatment reached only 26 ± 1.3% and 20 ± 1.0%, respectively. As expected, the capability of GSH to scavenge the excess ROS secreted by neutrophils and macrophages during the inflammatory phase has been evidenced without any doubt. On day 18, in rats treated with Ch/HA/GSH composite membranes, complete healing was perceived (almost 100%), whereas in the Ch/HA group and control group, the values were lower, equaling 81 ± 4.1% and 57 ± 2.9%, respectively.

As observed, the Ch/HA-treated wound commenced very early to generate a thin layer of neo-epithelium with the great proliferation of fibroblast cells accompanied by low inflammatory reactions. These findings can be explained by the synergistic behavior of chitosan and HA to accelerate tissue restoration. Interestingly, the skin tissue of the wound loaded with the Ch/HA/GSH membrane resurfaced by epidermis and keratinocyte layers with hair follicles and sebaceous glands, which point to the remarkable proliferation of fibroblast and keratinocyte cells to produce the connective tissue and collagen of the dermis layer in addition to the epidermis, referring to the superior influence of this membrane among the other groups. On day 18, the control group was in process of generating the epithelium layer, and the dermis became organized within the skin. Conversely, the sections of Ch/HA/GSH and Ch/HA-treated wounds exhibited denser epithelium layers compared to the previous investigations. However, wounds handled with Ch/HA/GSH composite membranes showed an entire reepithelialization, which is comparable to the distinctive structure of the healthy skin.

Although our previous articles demonstrated that the Ch/HA membrane, enhanced by agents such as edaravone and MitoQ, significantly contributes to wound healing in rats [36,148], the addition of GSH revealed how different free radical scavengers affect wound remodeling.

Generally, two chemical redox processes need to be considered in oxidative stress: The first case is the so-called “SET”—an endogenous essential constituent, e.g., of the R’ type, if it provides an oxidant, i.e., for example, a radical HO^●^, RO^●^, ROO^●^, ArO^●^, ArOO^●^ in an aqueous milieu, one e^−^_(aq)_ due to the ionic environment, the resulting anion takes a hydrogen cation from H_3_O^+^ (H_3_O^+^ → H_2_O + H^+^), resulting in the generation of a non-functional ^●^R′. In the second case, the so-called “HAT” endogenous donor R″-H provides the oxidant with a hydrogen atom (^●^H) and thus itself converts into ^●^R″. Nature maintains healthy life with multiple protective mechanisms. It has equipped phylogenetically younger organisms with a simple tripeptide—GSH—which provides one hydrogen atom to the oxidant and itself converts into oxidized GSSG. The resulting GSSG is converted back to GSH using glutathione disulfide reductase. Ergothioneine seems to sufficiently protect phylogenetically older organisms, who face stronger cosmic radiation rather than today’s high concentration of O_2_ in the atmosphere.

### 5.2. l-(+)-Ergothioneine

l-(+)-Ergothioneine, (S)-α-carboxy-*N,N,N*-trimethyl-2-mercapto-1H-imidazole-4-ethanaminium, C_9_H_15_N_3_O_2_S, molecular weight 229.3 Da, is a non-toxic bioactive molecule [156].

l-(+)-Ergothioneine is tautomeric and in neutral aqueous solutions exists predominantly in the thione form (cf. Figure 8), which may account for EGT’s resistance to autoxidation.

The only organisms known to synthesize EGT are mycobacteria belonging to the genus *Actinomycetales* and non-yeast-like fungi, which include members of the divisions *Basidiomycota* and *Ascomycota*. These microbes synthesize EGT from l-histidine via the intermediate hercynine—a betaine of histidine, then the enzyme EanB catalyzes the synthesis of l-(+)-ergothioneine directly from hercynine in the presence of a sulfur donor under anaerobic conditions [157]. In 2005, Gründemann et al. [158] and other researchers identified a selective organic cation transporter (OCTN1) and demonstrated that this protein distributes EGT to all key tissues, including the human brain, digestive tract, liver, heart, spleen and kidney. OCTN1 expression is increased at sites of tissue damage, and the subsequent increased EGT levels lead to cytoprotection and antioxidant properties [159,160,161,162,163].

Some questions remain to be solved, such as:

- Is EGT a self-acting and significant antioxidant in vivo [164,165]?
- Is it a “longevity vitamin” due to its cytoprotection [166]?

**Figure 11:**
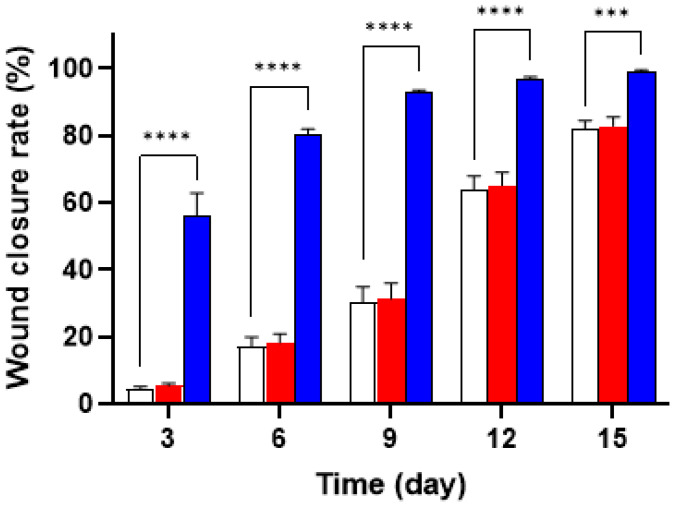
Wound closure rate vs. time in the ischemic rabbits: the untreated group (control, white), the group treated with the Ch/HA) membrane (red), the group treated with the Ch/HA/EGT membrane (blue). Data are presented as means ± SD, *n* = 6. Statistical analysis was performed using two-way ANOVA followed by Tukey’s multiple comparison test, *** *p* ≤ 0.001, **** *p* ≤ 0.0001 compared with the control group, *n* = 6 [38].

Figure 11 illustrates wound closure rate in ischemic male rabbits (2.5 ± 0.5 kg) during their treatment with the Ch/HA/EGT membrane (blue) and comparison with the Ch/HA membrane (red) and the untreated rabbits (control, white).

The Ch/HA/EGT membrane acting during the first three days showed the highest protective efficacy. On day 3, the untreated skin wounds and the wounds treated with Ch/HA membranes healed up to 5 and approx. 30%, respectively. The highest efficacy of the Ch/HA/EGT composite membrane was observed on days 6 and 9, reaching 80.3 ± 3.7% and 92.6 ± 2.1% wound closure rate, respectively. On day 9, the treatment with EGT resulted almost in a complete wound closure. Subsequently, on days 12 and 15, a similar efficacy of Ch/HA membranes was observed. On day 15, the wound closure rates reached almost 100%. On the other hand, the wound closures in the control group reached only 81.8 ± 6.6%.

Our study is the first, where EGT has been explored as a component of wound dressings used for the treatment of injured skin in ischemic rabbits and is the subject of a recent patent application [112]. The invention concerns self-associating biopolymers comprising a polyanionic (high-molecular-weight HA) and a polycationic biopolymer (chitosan), which together form a carrier for the cytoprotectant—l-(+)-ergothioneine—loosely incorporated into the carrier. The carrier, composed of HA and chitosan in a ratio that ensures that the carrier has a little surplus of negative charge, loosely binds EGT since its polarity is positive, i.e., opposite to the polarity of the carrier.

## 6. Conclusions

This review emphasizes a remarkable potential of chitosan–hyaluronan composite membranes as smart biomaterial platforms for the controlled delivery of antioxidants to skin wounds. By integrating selected antioxidants of both single-electron transfer and hydrogen-atom transfer mechanisms, including phosphatidylcholine dihydroquercetin, glutathione, l-(+)-ergothioneine, and mitochondria-targeted mesylate mitoquinone, these biopolymer systems demonstrated sustained release behavior, effective ROS scavenging, and superior wound healing efficacy.

The remarkably accelerated reepithelialization and almost complete wound closure observed in vivo confirm that the chitosan/hyaluronan membranes function not merely as passive dressings but as therapeutic matrices capable of modulating the redox environment of injured tissue. Their biocompatibility, moisture retention, and ability to maintain local redox balance position these materials among the most promising next-generation wound dressings.

## 7. Future Perspectives

The future development of antioxidant-enriched chitosan/hyaluronan wound dressings should follow a mechanistically guided and clinically informed strategy that respects the dual biological role of reactive oxygen species. The chitosan/hyaluronan platform itself already provides a biologically active baseline, improving wound healing compared to gauze itself, and thus represents a robust foundation for further functionalization.

The aim will be to introduce embedded redox- or pH-sensitive indicators into chitosan/hyaluronan membranes to create smart dressings for the treatment of skin diseases, such as acute surgical wounds, chronic diabetic ulcers, and ischemic and radiation-induced wounds. Priority should be given to hydrogen atom–transferring antioxidants, such as ergothioneine and glutathione, as safe and physiologically compatible baseline additives. These compounds effectively neutralize highly reactive species without disrupting redox signaling pathways essential for tissue regeneration. Their use as freely diffusible components of chitosan/hyaluronan membranes appears particularly suitable for long-term or extensive wound treatment.

For conditions of severe or persistent inflammation, a higher level of intervention may be introduced through chemically fixed electron-transferring antioxidants. By employing, e.g., pH-sensitive or even enzymatically cleavable spacers, these antioxidants could remain inactive under mild inflammatory conditions and become released only when pathological enzyme activity signals excessive oxidative stress. Such hierarchical activation would minimize systemic exposure while preserving therapeutic potency.

Future systems should aim to integrate both hydrogen atom transfer and set electron transfer antioxidants within a single adaptive platform, allowing smart control of antioxidant activity. This strategy is based on how redox defense systems work in the body and strikes a good balance between safety and effectiveness. Beyond material design, translational progress will require systematic evaluation of antioxidant release kinetics, local versus systemic exposure, and scalability under GMP-compatible manufacturing conditions. Stratification of wound types and patient oxidative status may further enable personalized membrane formulations tailored to specific clinical scenarios.

Ultimately, antioxidant-enriched chitosan/hyaluronan membranes should evolve toward responsive, biologically informed materials that do not merely cover wounds but actively communicate with the healing tissue. Such wound dressings have the potential to redefine therapeutic standards in chronic wound management, regenerative medicine, and redox pharmacology.

## Figures and Tables

**Table 1:** The standard one-electron reduction potential of the redox reaction of ROS, ascorbyl anion radical, and cupric ion [33].

Species ^a^	One-Electron Redox Couple	E°’/mV at pH 7
Hydroxyl radical	HO^●^, H^+^/H_2_O	+2310
Aliphatic alkoxyl radical	RO^●^, H^+^/ROH	+1600
Aliphatic peroxyl radical	ROO^●^, H^+^/ROOH	+1000
Hydrogen peroxide	H_2_O_2_, H^+^/H_2_O, HO^●^	+320
Ascorbyl anion radical	Asc^●−^, H^+^/AscH^−^	+282
Cupric ion	Cu(II), e^−^_(aq)_/Cu(I)	+159

^a^ The listed redox couples are selected from those free radical reactions that are of interest to the medicinal sciences. The greater positive value of the standard one-electron reduction potential (E°’) of free radicals indicates the greater oxidizing property of the given species. The free ^●^OH radical in aqueous media has the highest affinity to trap [H^+^ + e^−^_(aq)_], i.e., one hydrogen radical (H^●^). In other words, the free hydroxyl radical in contact with a biologically important compound by abstracting one hydrogen radical (hydrogen atom) turns into a molecule of water and usually yields a destructed bio-structure.

**Table 2:** Antioxidative properties of compounds investigated for their ROS scavenging properties using the WBOS.

Compound ^a^	Oximetry Arrangement		Viscometry Arrangement		Comment(s)	Refs.
	I.	II.	I.	II.		
Bucillamine	+++ ^b^	+++	+++	+++		[35]
Captopril	+++	+++	+++	++		[34]
Dithiotreitol	+++	++	+++	+++		[35]
Edaravone			+++	+++		[36]
l-(+)-Ergothioneine (EGT) ^c^	+++	+++	+++	+++	Reductive chelator of Cu^2+^ [37]	[38]
Glutathione	+++	+++	+++	+++		[39]
l-(+)-Hercynine			- ^d^	-		[38]
l-(+)-Histidine			+++	-		[38]
Levamisole	-	-	-	-		[34]
MnTE-2-PyP^5+^	prooxidant, strong					[40]
MnTnBuOE-2-PyP^5+^	prooxidant, mild					[40]
MnTM-4-PyP^5+^			+++	+++		[40]
Nor-methyl-l-ovothiol A	++	++	++	++		
Nor-trimethyl-ergothioneine)/ l-2-thiol-histidine	++	+++	+++	+++		
S-methyl-l-ergothioneine	+	+	+	+		
Tiopronin	+++	+++	+++	+++		[34]

^a^ Radiation and xenobiotics should be classified (cf. Figure 1) as significant actors, which can generate ROS by (i) radiation converting water molecules in the skin and (ii) biotransformation of xenobiotics—the chemical substances which are not naturally present within the organism. Xenobiotics also cover substances that are present in much higher concentrations than those usually occurring. They all may be grouped among food additives, drugs, environmental pollutants, carcinogens, pesticides, etc. ^b^ Effectivities scale, from most to less (+++ to +) to scavenge ROS. Compounds marked “+++” are appropriate for incorporation into Ch/HA membranes for wound healing. ^c^ Although Motohashi et al. [37] claimed that EGT forms complexes with copper ions by using X-band electron paramagnetic resonance, it has been demonstrated that the effect of EGT is reductive, producing cuprous ions. Yet, these ions do not trigger any Fenton-like generation of either superoxide anion radical or hydrogen peroxide, most probably due to a great excess of ascorbate antioxidative action using WBOS [38]. ^d^ Compounds ineffective against ROS.

**Table 3:** Physico-chemical and pharmacological effects of quercetin with its three derivatives (two are *O*-glycosides) and two phosphatidylcholine derivatives as active/antioxidative principles.

Polyphenol Sub-Class	Compound; Molar Mass [Da]	Solubility in Water [mg/mL]	Solubility in Ethanol [mg/mL]	logP	Actions	Refs. Comments
Flavonoids/Flavonols	Quercetin ^a^ 302.24	0.01	Very soluble	≈1.5	Antibacterial, anti-diabetic, anti-inflammatory, antineoplastic, and antioxidative agent, chelator, geroprotector, radical scavenger.	[122]
	Quercetin 3-*O*-glucoside/Isoquercetin/Hirsutrin ^b^ 464.38	0.095	NA	0.4	Antibacterial, anti-inflammatory, antineoplastic, antioxidative, anti-proliferative, and antipruritic agent, chelator, geroprotector, inhibitor of cell oxidative damage, scavenger of free radicals.	[122,127]
	Quercetin- 3-*O*-rutinoside/Rutin ^b^ 610.52	0.125	5.5	−1.3	Anti-carcinogenic, anti-diabetic, anti-inflammatory, and antioxidative, cardioprotective agent, decreases capillary fragility, food antioxidant, geroprotector, hepatoprotective, nephroprotective, neuroprotective agent.	[121,128]
	Dihydroquercetin/Taxifolin ^a^ 304.25	slight	≈3	0.95	Antibacterial, antihyperlipidemic, anti-inflammatory, antimycotic, antineoplastic, cardioprotective, and hepatoprotective agent, inhibitor of cellular melanogenesis, scavenger of free radicals.	[122,129] Four stereoisomers exist.
	Phosphatidylcholine quercetin ^c^ 913.69	Dispersible	Soluble	NA	Corresponding to those actions of quercetin.	[123,129]
	Phosphatidylcholine dihydroquercetin ^c^ 917.72	Dispersible ^d^	Soluble	NA	Corresponding to those actions of taxifolin.	[123,129]

^a^ The low water solubility of flavonoids/flavonols, when loaded into a hydrophilic skin-wound dressing, can be solved by the agent(s) host-guest complexation, within, e.g., 2-hydroxypropylated-β-cyclodextrin cavity {(2HP-β-CD, [130]; cf. also: [131,132,133]}. ^b^ Quercetin-*O*-glycosides are dispersed in aqueous solutions as micelles. Such *O*-glycosidic micelles can also serve as solubilizers of water-insoluble polyphenols such as quercetin with inner space filled with liposoluble quercetin, while the outer micelle polar sphere will be formed from 3-*O*-glucoside or 3-*O*-rutinoside. ^c^ Phosphatidylcholine preparations form liposomes, whose solubility in polar solvents is governed by phosphatidyl moieties. ^d^ Phospholipid derivatives of polyphenols, e.g., the prepared phosphatidylcholine dihydroquercetin according to zwitterionic phosphatidylcholine, were easily dispersed into the self-associating Ch/HA matrix serving as skin-wound dressings. The Ch/HA matrix is an appropriate reservoir for releasing the antioxidant agent or drug onto the skin wounds. NA—not available.

## Data Availability

The data presented in this study is openly available.

## Supplementary Materials

The following supporting information can be downloaded at: https://www.mdpi.com/article/10.3390/pharmaceutics18050603/s1, Table S1. Differences between HAT and SET mechanism. Table S2. Some selected trials treating diseases from a redox perspective. References [167,168,169,170,171,172,173,174,175,176,177,178,179,180,181,182,183,184,185,186,187] are cited in Supplementary Materials.

## Abbreviations

Ch	Chitosan
ECM	Extracellular matrix
EGT	Ergothioneine
ER	Endoplasmic reticulum
GSH	Glutathione
GSSG	Oxidized glutathione
HA	Hyaluronan
HAT	Hydrogen atom transfer
MitoQ	Mitoquinone mesylate
MTA	Mitochondrially-targeted antioxidant
NAC	*N*-Acetylcysteine
NADPH	Nicotinamide adenine dinucleotide phosphate
NAG	*N*-Acetylglucosamine
OCTN1	Organic cation transporter N1
PCDQ	Phosphatidylcholine dihydroquercetin
ROS	Reactive oxygen species
SET	Single electron transfer
SOD	Superoxide dismutase
WBOS	Weissberger biogenic oxidative system
