# Supplementary material for: Smart Release of the Antioxidant from Chitosan-Hyaluronan Reservoir in Skin Wound Healing
Source: Pharmaceutics. 2026 May 14;18(5):603. doi: 10.3390/pharmaceutics18050603 (PMC13210664; doi:10.3390/pharmaceutics18050603)
Supplement: Supplementary file 1 [file pharmaceutics-18-00603-s001.zip › pharmaceutics-4267406-supplementary.pdf]

Table S1. Differences between HAT and SET mechanism.

Characteristics	HAT (Hydrogen Atom Transfer)	SET (Single Electron Transfer)	Refs.
Basic mechanism	Transfer of a hydrogen atom ($H^{\bullet}$ = proton + electron) from antioxidant to radical	Transfer of a single electron (e^{-}) from antioxidant to radical	[1,2]
Biological relevance	Important in lipid peroxidation inhibition	Important in redox reactions in aqueous systems	[3,4]
General reaction	$ArOH + R^{\bullet} \rightarrow ArO^{\bullet} + RH$	$ArOH + R^{\bullet} \rightarrow ArOH^{\bullet+} + R^{-}$	[5]
Intermediate species	Neutral radicals, e.g., C-centered radicals	Charged intermediates (radical cations/anions)	[6,7]
Key thermodynamic parameter	Bond dissociation enthalpy of X–H bond	Ionization potential / redox potential	[1,8]
Main driving factor	Ability to donate hydrogen atom	Ability to donate electron	[1]
pH dependence	Independent of pH	Strongly pH-dependent (deprotonation enhances electron transfer)	[9]
Products formed	Neutral molecule and antioxidant radical ($ArO^{\bullet}$)	Radical cation ($ArOH^{\bullet+}$) and reduced species	[5]
Reaction speed	Generally fast (kinetically controlled)	Often slower, may depend on equilibrium	[9]
Solvent effect	Non polar, weak polar solvents	Strong dependence (ionic intermediates stabilized in polar solvents)	[1]
Type of transfer	Concerted (proton and electron together)	Stepwise (electron first, often followed by proton transfer)	[10,11]

Table S2. Some selected trials treating diseases from a redox perspective[a].

Antioxidant	Indication	Targeted mechanism	Title	Phase	Code	Refs.
Captopril	Skin Laceration		Healing of Skin Lacerations in Ischemic Ears of Rabbits	Pre-clinical	2/0019/19[b]	[12]
Edaravone	Skin Laceration		Healing of Skin Lacerations in Ischemic Ears of Rabbits	Pre-clinical	2/0065/15[b]; 2/0019/19[b]	[13,14]
Edaravone	Alzheimer's Disease	Nrf2 activator	Alzheimer Study Using Oral Edaravone	II	NCT 05323812	[15]
L-(+)-Ergothioneine[c]	Kidney Failure		Repletion of Ergothioneine in Patients With Kidney Failure	I/II	NCT 06487546	[16]
L-(+)-Ergothioneine	Skin Laceration		Healing of Skin Lacerations in Rats and Ischemic Ears of Rabbits	Pre-clinical	2/0019/19[b]	[17]
L-(+)-Ergothioneine	Radiation-Induced Skin Injury		Ergothioneine-Sodium Hyaluronate Dressing Protecting against Radiation-Induced Skin Injury	Pre-clinical	-	[18]
Flavonoids	Colorectal Cancer	Nrf2 activator	Dietary Bioflavonoid Supplementation for the Prevention of Neoplasia Recurrence	II	NCT 00609310	[15]
Glutathione	Skin Laceration		Healing of Skin Lacerations in Rats	Pre-clinical	2/0019/19[b]	[19]
Glutathione	Alzheimer's Disease	Buffering ROS	Glutathione, Brain Metabolism and Inflammation in Alzheimer's Disease	Early I	NCT 04740580	[15]
Glutathione	Parkinson's Disease	Buffering ROS	Glutathione in the Treatment of Parkinson's Disease	II	NCT 01177319	[15]

Glutathione	Post Burn Facial Hyperpigmentation		Glutathione Mesotherapy in Post Burn Facial Hyperpigmentation	II	NCT07263841	[20]
Lipoic Acid	Heart Failure	Nrf2 activator	Clinical Study of Lipoic Acid on Ischemic Heart Failure	IV	NCT 03491969	[15]
Lipoic Acid	Non-Alcoholic Fatty Liver Disease (NAFLD)	Nrf2 activator	Effect of Alpha Lipoic Acid on NAFLD	IV	NCT 04475276	[15]
Melatonin	Type 2 Diabetes Mellitus	Nrf2 activator	Melatonin's Effects on the Treatment of Diabetes Mellitus	Early I	NCT 02691897	[15]
Mitoquinone mesylate (MitoQ)	Chronic Hepatitis C	Mitochondrial oxidative stress	Trial of MitoQ for Raised Liver Enzymes Due to Hepatitis C	II	NCT 00433108	[21]
MitoQ	Skin Laceration		Healing of Skin Lacerations in Rats and Ischemic Ears of Rabbits	Pre-clinical	2/0065/15[b]	[22]
MitoQ	Frostbites		MitoQ Supplementation on Skin Blood Flow in the Cold	N/A	NCT06784531	[23]
N-Acetylcysteine (NAC)	Gaucher's and Parkinson's Disease	Buffering ROS	Intravenous NAC for the Treatment of Gaucher's and Parkinson's Disease	I	NCT 01427517	[15]
NAC	Parkinson's Disease	Buffering ROS	Repeated-Dose Oral NAC for the Treatment of Parkinson's Disease	II	NCT 02212678	[15]
NAC	NAFLD	Buffering ROS	NAC and Patients with NAFLD	III	NCT 05589584	[15]
NAC	Heart Failure with Chronic Renal Failure	Buffering ROS	NAC in Heart Failure with Coexistent Chronic Renal Failure	III	NCT 00532688	[15]
NAC	Head and Neck Neoplasms	Buffering ROS	Evaluation of the Use of NAC Attenuating Cisplatin-Induced	IV	NCT 02241876	[15]

			Toxicities by Oxidative Stress in Head and Neck Cancer Patients			
NAC	Pressure ulcers		Topical treatment of non-healing pressure ulcers with NAC	Pre-clinical (case report)		[24]
Quercetin	Chronic Obstructive Pulmonary Disease (COPD)	Nrf2 activator	Beneficial Effects of Quercetin in COPD	II	NCT 06003270	[15]
Phosphatidylcholine dihydroquercetin	Skin Laceration		Healing of Skin Lacerations in Rats	Pre-clinical	2/0019/19[a]	[25]
Polyphenol(s) Green Tea	Parkinson's Disease	Nrf2 activator	Efficacy and Safety of Green Tea Polyphenol in *De Novo* Parkinson's Disease Patients	II	NCT 00461942	[15]
Vitamins C, E, and Ferulic acid[d]	Skin Quality		Protection of the Skin from UV Radiation	Clinical trial	-	[26]
Tiopronin	Skin Laceration		Healing of Skin Lacerations in Ischemic Ears of Rabbits	Pre-clinical	2/0019/19[b]	[27]

[a]Boosting the biological antioxidant system is a common strategy in antioxidant therapy [28,29].

[b]The Scientific Grant Agency of the Ministry of Education, Research, Development and Youth of the Slovak Republic and the Slovak Academy of Sciences.

[c]There is (only) one publicly registered clinical trial focused on L-(+)-ergothioneine.

[d]There is (only) one clinical trial that involves an antioxidant loaded onto skin and is currently in Phase II.

References

[1] Lewandowska, H.; Swisłocka, R.; S. Orzechowska, S. Towards the development of effective antioxidants – The molecular structure and properties – Part 2. *Molecules* **2026**, *31*,720.

[2] Platzer, M.; Kiese, S.; Herfellner, T.; Schweiggert-Weisz, U.; Miesbauer, O.; Eisner, P. Common trends and differences in antioxidant activity analysis of phenolic substances using single electron transfer based assays. *Molecules* **2021**, *26*, 1244.

[3] Munteanu, I.G.; Apetrei, C. Analytical methods used in determining antioxidant activity: A Review. *Int. J. Mol. Sci.* **2021**, *22*, 3380.

[4] Dehdari, A.; Poon J.-F.; Pratt D.A. Intramolecular hydrogen atom transfer enables hydroperoxyl radical formation during the peroxidation of unsaturated lipids. *Am. Chem. Soc.* **2025**, *147 (45)*, 41897–41912.
[5] Torres-Osorio, V.; Urrego, R.; Echeverri-Zuluaga, J.J., López-Herrera, A. Oxidative stress and antioxidant use during in vitro mammal embryo production. Review. *Rev. Mex. Cienc. Pecu.* **2019**, *10(2)*, 433–459.
[6] Funes-Ardoiz, I.; Garrido-Barros, P. Controlling selectivity of hydrogen atom transfer (HAT) in photoredox catalysis. *Chem. Catalysis* **2024**, *4*, 1−4.
[7] Mao, B.; Yan, J.; Wei, Y.; Shi, M. Hydrogen atom transfer promoted by carbon-centered biradicals via energy transfer catalysis. *Acc. Chem. Res.* **2025**, *58(13)*, 2028–2045.
[8] Ningjian, L.; Kitts, D.D. Antioxidant property of coffee components: Assessment of methods that define mechanisms of action. *Molecules* **2014**, *19(11)*, 19180–19208.
[9] Gulcin I. Antioxidants and antioxidant methods: an updated overview. *Arch. Toxicol.* **2020**, *94*, 651–715.
[10] Capaldo, L.; Ravelli, D. Hydrogen atom transfer (HAT): A versatile strategy for substrate activation in photocatalyzed organic synthesis. *Eur. J. Org. Chem.* **2017**, 2056–2071.
[11] Bakheit, A.H.; Wani, T.A.; Al-Majed, A.A.; Alkahtani, H.M.; Alanazi, M.M.; Alqahtani, F.R.; Zargar S. Theoretical study of the antioxidant mechanism and structure-activity relationships of 1,3,4-oxadiazol-2-ylthieno[2,3-d]pyrimidin-4-amine derivatives: a computational approach. *Front. Chem.* **2024**, *12*, 1443718.
[12] Valachova, K.; Svik, K.; Biro, C.; Soltes, L. Skin wound healing with composite biomembranes loaded by tiopronin or captopril. *J. Biotechnol.* **2020**, *310*, 49–53.
[13] Tamer, T.M.; Valachová, K.; Hassan, M.A.; Omer, A.M.; El-Shafeey, M.; Eldin, M.S.M.; Šoltés, L. Chitosan/hyaluronan/edaravone membranes for anti-inflammatory wound dressing: In vitro and in vivo evaluation studies. *Mater. Sci. Eng. C Mater. Biol. Appl.* **2018**, *90*, 227–235.
[14] Valachová, K.; Švík, K.; Jurčík, R.; Ondruška, Ľ.; Biró, C.; Šoltés, L. Enhanced healing of skin wounds in ischemic rabbits using chitosan/hyaluronan/edaravone composite membranes: Effects of laponite, carbon and silver-plated carbon nanofiber fillers. *Chem. Pap.* **2023**, *77(4)*, 1835–1841.
[15] Li, B.; Ming, H.; Qin, S.; Nice, E.C.; Dong, J.; Du, Z.; Huang, C. Redox regulation: Mechanisms, biology and therapeutic targets in diseases. *Signal Transduct. Target Ther.* **2025**, *10(1)*, 72.
[16] Repletion of Ergothioneine in Patients With Kidney Failure, https://clinicaltrials.gov/study/NCT06487546, available on April 29, 2026.
[17] Valachová, K.; Švík, K.; Biró, C; Collins, M.N.; Jurčík, R.; Ondruška, L.; Šoltés, L. Impact of ergothioneine, hercynine, and histidine on oxidative degradation of hyaluronan and wound healing. *Polymers (Basel)* **2020**, *13(1)*, 95.
[18] Tian, X.; Guo, J.; Gu, C.; Wang, H.; Wang, D.; Liao, Y.; Zhu, S.; Zhao, M.; Gu, Z. Ergothioneine-sodium hyaluronate dressing: A promising approach for protecting against radiation-induced skin injury. *ACS Appl. Mater. Interfaces*. **2024**, *16(23)*, 29917−29929.

[19] Tamer, T.M.; Hassan, M.A.; Valachová, K.; Omer, A.M.; El-Shafeey, M.E.A.; Eldin, M.S.M.; Šoltés, L. Enhancement of wound healing by chitosan/hyaluronan polyelectrolyte membrane loaded with glutathione: In vitro and in vivo evaluations. *J. Biotechnol.* **2020**, *310*, 103–113.
[20] Comparison of Effectiveness of Tranexamic Acid Mesotherapy Versus Glutathione Mesotherapy in Post Burn Facial Hyperpigmentation, https://clinicaltrials.gov/study/NCT07263841, available on April 29, 2026.
[21] Trial of MitoQ for raised liver enzymes due to hepatitis C, https://www.clinicaltrials.gov/study/NCT00433108, available online on April 29, 2026.
[22] Hassan, M.A.; Tamer, T.M.; Valachová, K.; Omer, A.M.; El-Shafeey, M.; Eldin, M.S.M.; Šoltés, L. Antioxidant and antibacterial polyelectrolyte wound dressing based on chitosan/hyaluronan/phosphatidylcholine dihydroquercetin. *Int. J. Biol. Macromol.* **2021**, *166*, 18–31.
[23] Researcher View | NCT06784531 | The Influence of Race and MitoQ Supplementation on Skin Blood Flow in the Cold | ClinicalTrials.gov, available online on April 29, 2026.
[24] Ozkaya, H.; Bahat, G.; Tufan, A.; Doğan, H.; Bilicen, Z.; Karan, M.A. Successful treatment of non-healing pressure ulcers with topical *N*-acetylcysteine. *J. Wound Care* **2015**, *12*, 606, 608‒611.
[25] Tamer, M.T.; Collins, M.N.; Valachova, K.; Hassan, M.A.; Omer, A.M.; Mohy-Eldin, M.S.; Svik, K.; Jurcik, R.; Ondruska, L.; Biro, C.; Albadarin, A.B.; Soltes, L. MitoQ loaded chitosan-hyaluronan composite membranes for wound healing. *Materials (Basel)* **2018**, *11(4)*, 569.
[26] Murray, J.C.; Burch, J.A.; Streilein, R.D.; Iannacchione, M.A.; Hall, R.P.; Pinnell, S.R. A topical antioxidant solution containing vitamins C and E stabilized by ferulic acid provides protection for human skin against damage caused by ultraviolet irradiation. *J. Am. Acad. Dermatol.* **2008**, *59(3)*, 418-425.
[27] Valachova, K.; Svik, K.; Biro, C.; Soltes, L. Skin wound healing with composite biomembranes loaded by tiopronin or captopril. *J. Biotechnol.* **2020**, *310*, 49–53.
[28] Guan, Q.; Du, C. Antioxidant nanozymes for prevention of diseased kidney from failure. *Kidney Int.* **2022**, *102(5)*, 961–963.
[29] Singh, N.; Sherin, G. R.; Mugesh, G. Antioxidant and prooxidant nanozymes: From cellular redox regulation to 1229 next-generation therapeutics. *Angew. Chem. Int. Ed. Engl.* **2023**, *62(33)*, e202301232.
